# Comparing Genetic *N_e_* Reconstructions Over Time With Long‐Time Wolf Monitoring Data in Two Populations

**DOI:** 10.1111/eva.70022

**Published:** 2024-10-17

**Authors:** Laia Pérez‐Sorribes, Pau Villar‐Yanez, Linnéa Smeds, Joachim Mergeay

**Affiliations:** ^1^ Department of Ecology and Evolution Estación Biológica de Doñana Seville Spain; ^2^ Universitat Politècnica de Catalunya Barcelona Spain; ^3^ Department of Ecology and Genetics Uppsala University Uppsala Sweden; ^4^ Research Institute for Nature and Forest Geraardsbergen Belgium; ^5^ Ecology, Evolution and Biodiversity Conservation KU Leuven Leuven Belgium

**Keywords:** conservation genetics, effective population size, metapopulation

## Abstract

Many methods are now available to calculate *N*_*e*_, but their performance varies depending on assumptions. Although simulated data are useful to discover certain types of bias, real empirical data supported by detailed known population histories allow us to discern how well methods perform with actual messy and complex data. Here, we focus on two genomic data sets of grey wolf populations for which population size changes of the past 40–120 years are well documented. We use this background to explore in what detail we can retrieve the known population history from these populations, in the light of pitfalls relating to population history, sampling design and the change in the spatial scale at which *N*_*e*_ is estimated as we go further back in time. The Scandinavian wolf population was founded in the early 1980s from a few individuals and has gradually expanded up to 510 wolves. Although the founder event of the Scandinavian population was detected by GONE, the founding effective population size was strongly overestimated when the most recent samples were used, but less so when older samples were considered. Nevertheless, the present‐day *N*_*e*_ corresponds to theoretical expectations. The western Great Lakes wolf population of Minnesota is the only population in the contiguous United States that persisted throughout the 20th century, surviving intense persecution. We found a good concordance between the estimated *N*_*e*_ and trends in census size data, but the reconstruction of *N*_*e*_ clearly highlights the difficulty of interpreting results in spatially structured populations that underwent demographic fluctuations.

## 1 Introduction

In an age of ecological turmoil and biodiversity decline, the need for monitoring biodiversity in all its facets, detect and record changes, and discover the reasons for those changes, has never been greater. Genetic aspects of biodiversity have long been acknowledged, but all too long merely as lip service (Laikre 2010; Laikre et al. 2010, 2020). Recently, however, explicit genetic indicators have been recognised for the United Nations' Global Biodiversity Framework, which includes a headline indicator that focuses on the effective size of populations, Headline Indicator A4. Meanwhile, methods are being tested and put into place to aid in monitoring of these indicators (Hoban et al. 2023; Mastretta‐Yanes et al. 2023).

Monitoring populations genetically is challenging, however. Even in countries considered at the forefront of genetic diversity monitoring, genetic monitoring programmes are still few and far apart (Moussy et al. 2022; Pearman et al. 2024). However, the growing number of population genomic studies and genomic resources for non‐model organisms are providing an incredible volume of public data, which can be harnessed for biodiversity research. In combination with new analytical tools for *N*_*e*_ estimation (e.g., Santiago et al. 2020), this allows the reconstruction of effective population size trends across timescales relevant to conservation, even for species devoid of long‐term sampling archives or monitoring programmes. The effective size of a population reflects how fast it loses genetic diversity compared to an ideal theoretical Wright–Fisher population and is therefore a key indicator in conservation biology. In general, the effective size is much smaller than the total number of individuals, due to large variance in the contribution of individuals to the next generation (Waples 2022). Estimating *N*_*e*_ trends allow us to quantify the impact of genetic drift and inbreeding in a population and to have a general idea of a baseline for conservation goals. Numerous approaches can be employed to estimate contemporary and historical *N*_*e*_, through the analysis of genetic markers (Nadachowska‐Brzyska, Konczal, and Babik 2022).

When an individual is genetically analysed, we are also sampling two half genomes of the parents, four quarter genomes of the grandparents and so on. Each fraction of these ancestor's genomes contains information about the history of the population, and we can use that, in principle, to make inferences on past demographic changes. In effect, even single genomes have been used to reconstruct population histories up to thousands of generations ago (e.g., Cuevas‐Caball et al. 2022). However, the spatial scale at which inferences are being made, and ancestors are drawn from the total distribution, also increases in spatially structured populations the further we go back in time (Novo et al. 2023a), making interpretation of reconstructed *N*_*e*_ values challenging. Even in demographically stable metapopulations, this leads to a spurious signal of population decline from the past to the present in the respective subpopulations. This is nothing but an artefact of population structure and the increasing spatial diffusion of the genetic material of an individual among its ancestors: Every generation back in time, our cumulative ancestors are spread out over a larger area until eventually every living member of that species at that time was either an ancestor of every contemporary individual or of none (Rohde, Olson, and Chang 2004). As a consequence, the *N*_*e*_ reconstructed is also drawn from a larger geographic area the further we go back in time, even though the samples were taken from a small area representing only a subpopulation or a single genetic neighbourhood (Novo et al. 2023a).

### 1.1 Estimating *N*_*e*_ From Linkage Disequilibrium (LD)

In an ideal Hardy–Weinberg population, alleles at unlinked neutral loci are inherited completely independently due to recombination at meiosis. When the population is finite, however, genetic drift and inbreeding are inevitable, and this will lead to covariance of alleles at unlinked loci through identity by descent. This gametic disequilibrium (typically called linkage disequilibrium) is thus a function of the effective size of the population. When loci are not independent, the linkage also depends on the physical distance on the chromosome, and therefore the recombination rate by crossing over at meiosis (Hill 1981). Hayes et al. (2003) showed that LD between pairs of loci at different genetic distances provides differential information on *N*_*e*_ at different time points in the past, which allows us to trace back *N*_*e*_ in time across tens to hundreds of generations. Santiago et al. (2020) refined this principle further and provided a software tool (GONE) for analysis of genomic data sets.

Importantly, the LD method assumes that the population is sampled randomly and experiences random mating across the entire population. In practice, few populations conform to this latter assumption, and there is typically a gradual spatial genetic structure across individuals of the population (isolation by distance), caused by limits to dispersal across the extent of the entire population. Wright (1946) described this through a ‘genetic neighbourhood’: As long as individuals are sampled from a circle with radius two times the average dispersal distance (the breeding window), the individuals can be considered panmictic. When a sample of genotypes from within a breeding window is analysed with LD, the *N*_*e*_ represents the effective size of the neighbourhood (Neel et al. 2013). When individuals are sampled from a larger area, however, the resulting LD *N*_*e*_ estimate is smaller than expected owing to the genetic structure that increases LD on top of LD caused by genetic drift. Samples that are composed of individuals from distinct subpopulations or neighbourhoods in a continuous population will underestimate the true *N*_*e*_ when LD is used (Neel et al. 2013). A similar effect of mixture LD occurs when samples from different ages or cohorts are combined (Waples, Antao, and Luikart 2014).

GONE (Santiago et al. 2020) has been tested extensively in silico (Novo, Santiago, and Caballero 2022; Novo et al. 2023a; Reid and Pinsky 2022; Santiago et al. 2020; Saura et al. 2021), and also experimental populations have recently been used to test its robustness (Novo et al. 2023b). The method has meanwhile been applied to a variety of species and populations of domesticated and wild species (e.g., Alvarez‐Estape et al. 2023; Iijima et al. 2023; Magnier et al. 2022; Wersebe and Weider 2023). The advantage of using domesticated species is that their history is often known in great detail, which allows testing the accuracy of the method, and test the sensitivity of specific assumptions. However, there is also a need to evaluate GONE with more complex data sets of natural populations for which census size trends are known (e.g., Kessler and Shafer 2024). Here, we test its efficacy in two wolf populations with well‐known population histories.

The grey wolf (*Canis lupus*) is a flagship species and keystone top predator across much of the northern hemisphere. They are among the best monitored species worldwide (Hindrikson et al. 2017) and a plethora of genetic and genomic resources are available for the wolf and its derived human friend, the dog (e.g., Tang et al. 2019). In this study, we use two open access data sets from wolf populations with different past histories where census population size (*N*_*c*_) of the past 40–120 years is known and for which either high‐density SNP genotypes or WGS data are available, allowing us to test how well GONE (Santiago et al. 2020) is capable of reconstructing known demographic changes over time in real populations with complex histories and with known violations of underlying assumptions.

## 2 Methods

### 2.1 Data Sets

We selected two grey wolves (*Canis lupus*) genomic open access data sets from which great detail census size (*N*_*c*_) information is available (number of individuals and packs) as a result of decades of monitoring. These populations are genetically clearly distinct and geographically delimited and exhibit markedly divergent historical trajectories, as detailed in the subsequent sections.

#### 2.1.1 Dataset 1: Western Great Lakes Population, Minnesota, North America

**FIGURE 1:**
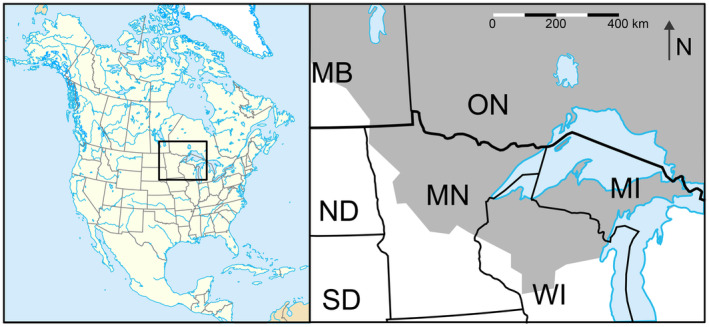
Detailed comparison of *N*_*e*_ (black line) for the Western Great Lakes wolves reconstructed since 1900 (25 generations) and inferred total population size (*N*_tot_) (grey line). Total population size was deduced from Stenlund (1955) and Fuller et al. (1992) up to 1976 (open circles plus minimum and maximum estimates) and reproduced from monitoring data from Erb and Humpal (2022) from 1978 to the present (full circles). We assumed a generation interval of 4 years.

The Great Lakes region of North America features a complex network of interconnected freshwater lakes. Recognised for its ecological diversity, this area supports a rich boreal ecosystem that has been a natural habitat for grey wolves since ancient times. Here, we focus on the wolf population of Minnesota (Figure 1), which has long been the core of the Western Great Lakes wolf population, and which is the only state in the contiguous United States where wolves were not exterminated. This population has fluctuated as a result of human interference, and these changes have been documented with varying levels of detail, which we can compare to *N*_*e*_ reconstructions.

Prior to settlement by Europeans, ‘all of Minnesota’ was inhabited by wolves (Stenlund 1955). Wisconsin, to the east, had an estimated 3000–5000 wolves (Wisconsin Department of Natural Resources 2024). Extrapolating this density (0.0175–0.030 ind/km^2^; Rutledge et al. 2010 apply 0.025–0.035/km^2^ in E‐Ontario) to Minnesota, the entire state likely had 4000–7000 wolves. The neighbouring region of Ontario also likely had thousands of wolves (Omand 1950). The first verified European presence was in the 17th century, which consisted of French fur traders and trappers (‘voyageurs’), with a focus on beaver pelts. Until 1850, most of the territory was inhabited by First Nations of the Ojibwe (N and E) and Dakota people (S and W), who were closely involved in the fur trade. The first permanent US settlement in the area was the military base of Fort Snelling in 1819. As native ungulates populations became depleted by unregulated hunting by European settlers (Stenlund 1955), wolves switched increasingly to livestock, and the human pressure on wolves increased (Wisconsin Department of Natural Resources 2024). By 1900, woodland caribou were extinct, and elk disappeared in the first half of the 20th century (Stenlund 1955). Bounty payments for killed wolves started in 1849, when Minnesota became part of the USA, and lasted until 1965 (Fuller et al. 1992). A similar bounty system was in place in Wisconsin, which led to the extinction of wolves by 1950. In North Dakota, wolves were already extinct by 1922. In SW‐Ontario bounty harvests, first started in 1793, varied from 1000 to 5000 wolves per year between Kenora and Thunder Bay (the area just north of Minnesota) between 1925 and 1931 (Omand 1950). By 1900, wolves were rare in the prairie region of south and west Minnesota, and deforestation had impacted large areas of the northern part (Stenlund 1955). Wolf densities remained relatively high in the forested areas of north and east Minnesota. Excluding the prairies of Minnesota, the remaining wolf habitat covered circa 70,000 km^2^. Applying the same conversion (0.017–0.030 wolves/km^2^), this yields a ball‐park estimate of 1700 (1200–2100) wolves around 1900 AD. The wolf range decreased further between 1915 and 1941, and high densities occurred only in the northern part of the state. Around 1950, Stenlund (1955) estimates that densities had halved even in densely forested areas, with a state‐wide population size of 450–700 wolves, concentrated in the northeast (Fuller et al. 1992). By this time, wolves were extinct in the contiguous United States except for northern Minnesota. Even after wolves were considered endangered in 1965, hunting was still allowed year‐round, with annual harvests in Minnesota around 200 wolves until 1973, on top of a predator control programme averaging 64 wolves per year. Considering that the population seemed stable, Fuller et al. (1992) concluded that this level of harvest mortality corresponded to a population size of 736–950 wolves in the early 1970s, assuming the maximum sustainable harvest rate is 28%.

Since 1978, there has been an official annual monitoring conducted by the Minnesota Department of Natural Resources. This census has been carried out by merging features of territory mapping with specific methods to ascertain the overall area of the state inhabited by wolf packs. The techniques utilised have seen minimal alterations throughout this period. Initially, surveys were conducted approximately every 10 years (1978, 1988, 1998), followed by more frequent assessments approximately every 5 years (2003, 2007, 2012) and annual counts since 2012 (Erb and Humpal 2022). Between 1978 and 1998, the population doubled from 1235 to 2445 wolves, and it has fluctuated around 2500 wolves ever since (MN DNR 2023). Following legal protection and subsequent population recovery in Minnesota, wolves recolonised Wisconsin in 1978, and subsequently also Michigan in 1989 (Michigan Department of Natural Resources 2023). They currently number circa 1000 wolves in 290 packs in Wisconsin (Wisconsin Department of Natural Resources 2023) and circa 650 wolves in Michigan since 2010 (Michigan Department of Natural Resources 2022). In North of Minnesota, wolves were never extirpated either, but little data exist on their demographic evolution. Given that a similar bounty system existed, and that wolves have always remained a game species in Ontario we can assume the population was similarly impacted.

Cronin et al. (2015) genotyped 20 great lakes wolves at 123,801 SNPs (llumina170K CanineBeadChip), sampled in 2012 and 2013 by the Minnesota Department of Natural Resources (Table S1). Samples were taken in the northern half of Minnesota, with the largest pairwise distance between samples of 450 km, approximately. Cronin et al. (2015) and Pilot et al. (2019) showed these wolves to be genetically homogenous and distinct from other North American wolf populations. Since the samples were all taken from a relatively small area (not much larger than the breeding window of wolves; Mergeay et al. in press) and from a single cohort, we can ascertain that assumptions of LD‐based methods (random sampling, random mating, single cohort) are unlikely to be strongly violated.

#### 2.1.2 Dataset 2: Scandinavian Peninsula Population, Europe

Scandinavian wolves currently belong to a small and highly inbred population (Kardos et al. 2018). After a steep 150‐year long decline due to human persecution (Flagstad et al. 2003), wolves were functionally extinct in Scandinavia in the 1960s. Legal protection followed in 1966 in Sweden and in 1972 in Norway (Wabakken et al. 2001). The closest source population was the Finnish–Karelian population (Chapron et al. 2014), but with a zero tolerance to wolves in N‐Finland, which continues up to this day, recolonisation of the Scandinavian peninsula was delayed. In 1978, a first reproduction event occurred in N‐Sweden but did not persist. A second litter was produced in S‐Sweden in 1983, but the population size never exceeded 1 pack until 1991, when an immigrant male sired a litter with one of the offspring of the first pair (Wabakken et al. 2001). All population growth until 2008 was the result of these three founders (Liberg et al. 2005; Vilà et al. 2003; Wabakken et al. 2001). As a result, the population became highly inbred (Kardos et al. 2018; Smeds and Ellegren 2022). It was not until 2008 and 2013 that new immigrants integrated into the population and reproduced in Scandinavia (Åkesson et al. 2022). As of 2022, approximately 40 immigrant wolves have been detected since 1991, only nine of which have contributed genetically (Åkesson et al. 2023). Between 2018 and 2022, the population size has fluctuated between 380 and 540 individuals, across 40–55 packs (Svensson et al. 2023).

Genotypes from the most contemporary available Scandinavian wolves were extracted from Kardos et al. (2018) and Smeds and Ellegren (2022), a high‐coverage (average 17.6×), whole genome sequencing data set. A total of 10,622,231 variant sites from 41 individuals from 2007 to 2014 were used to run GONE (see Table S2). We ran the analysis with all 41 individuals (including immigrants of 2008 and 2013) and without the four immigrant genotypes. In addition to this, we ran the analyses with samples from older cohorts (1983–1990, 1991–1998 and 1999–2006 with an average coverage of 33.8×, 33.1× and 25.8× respectively). We acknowledge that these samples were always drawn from just over one generation, but the published data do not allow us to discern between them, and the point is to illustrate the usefulness of open access data in the way they are archived.

### 2.2 Data Analysis

We reconstructed the recent *Ne* demographic history (< 100 generations) of the Great Lakes and Scandinavian wolf populations using GONE (Santiago et al. 2020). We ran 40 independent replicates with autosomal data using standard input parameters, with the average recombination rate set to 0.90 cM/Mb (Campbell et al. 2016; Pacheco et al. 2022). A complete workflow of the analyses carried out in GONE is available at https://github.com/villarpau/Wolf_Ne_reconstructions.

We compared the most recent *N*_*e*_ estimates for each cohort with expectations based on pedigree‐based simulations by Bruford (2015) for the Scandinavian wolf population, and with monitoring data from the Minnesota Department of Natural Resources, plus literature data to reconstruct population sizes prior to 1978.

## 3 Results and Discussion

### 3.1 Western Great Lakes Population, Minnesota, North America

**FIGURE 2:**
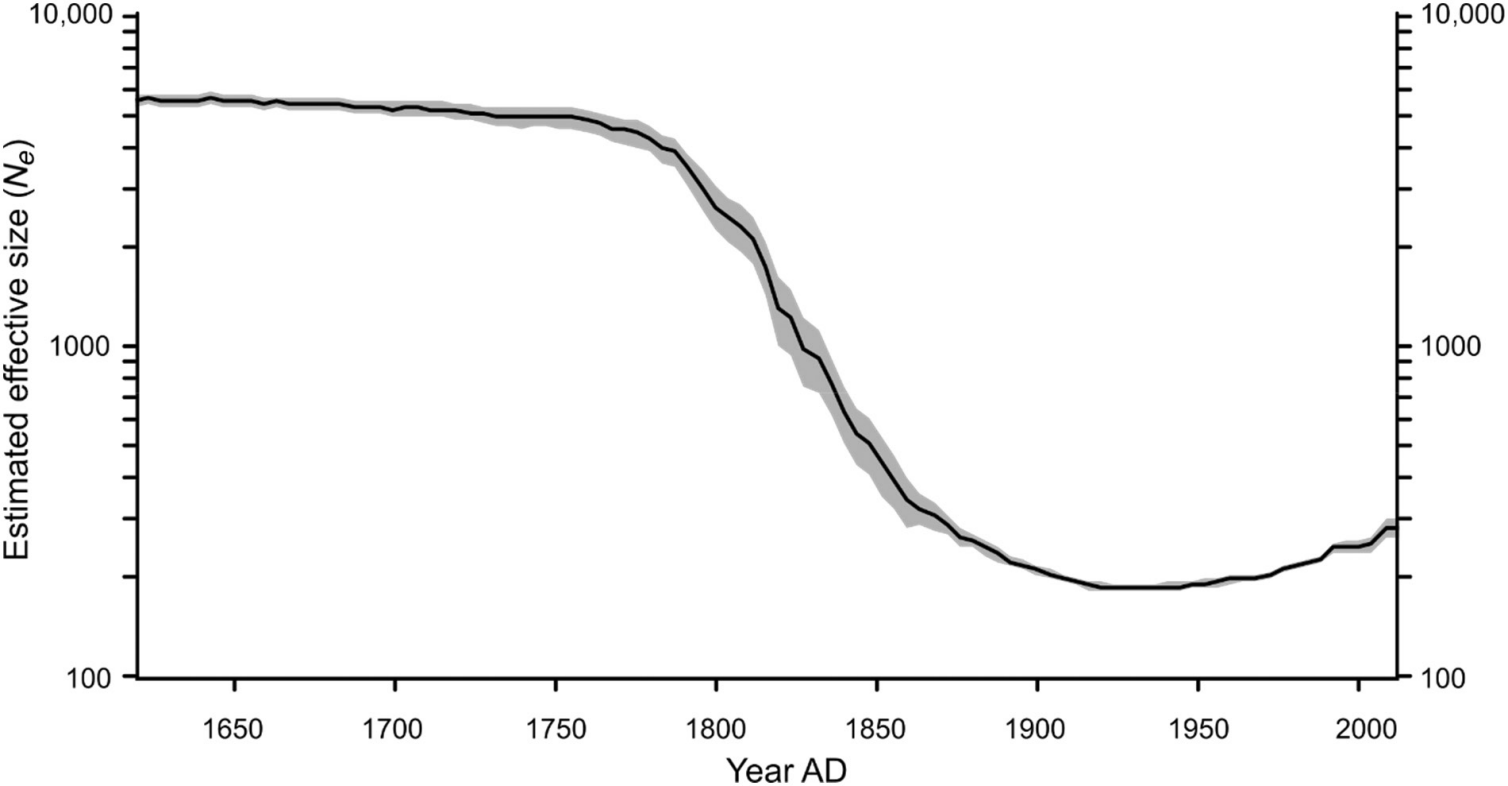
Reconstruction of *N*_*e*_ for the past 100 generations for the Minnesota wolf population. The shaded area represents the 95% confidence interval of the estimates obtained by running 40 replicates with 15 random individual combinations of 20 samples across 100 generations (4 years/generation).

**FIGURE 3:**
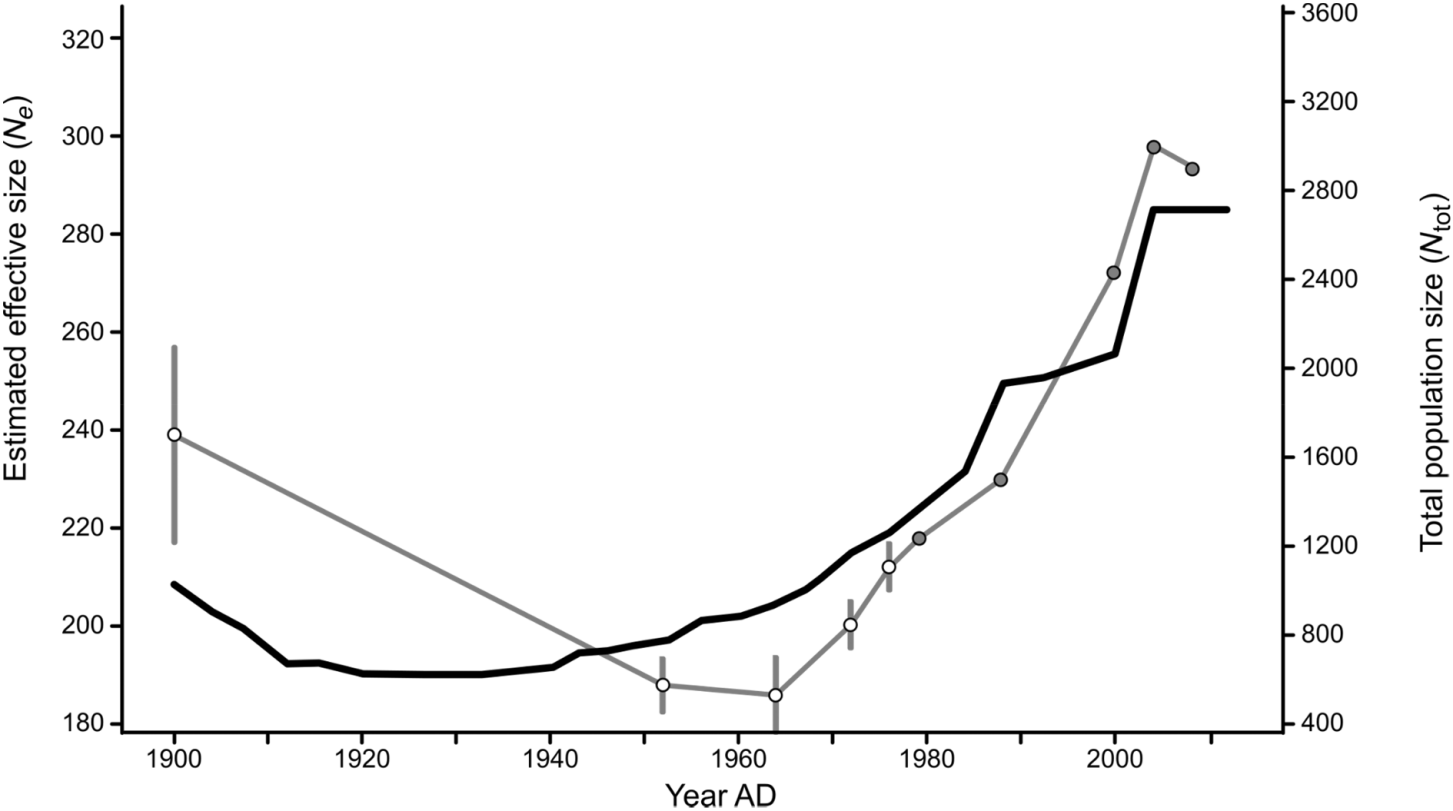
Detailed comparison of *N*_*e*_ (black line) for the Western Great Lakes wolves reconstructed since 1900 (25 generations) and inferred total population size (*N*_tot_) (grey line). Total population size was deduced from Stenlund (1955) and Fuller et al. (1992) up to 1976 (open circles plus minimum and maximum estimates) and reproduced from monitoring data from Erb and Humpal (2022) from 1978 to the present (full circles). We assumed a generation interval of 4 years.

The reconstructed *N*_*e*_ shows a marked drop from a stable effective size exceeding 5000 prior to European presence, to a minimum of 190 around 1930 AD (assuming 4 years per generation; Mech, Barber‐Meyer, and Erb 2016) (Figure 2). Focusing on the period 1900–2010 (the period covered by the demographic reconstruction), we see a good overall concordance between the census size and the effective size (Figure 3), even though the change is not entirely linear. Prior to 1900, however, the reconstructed *N*_*e*_ was an order of magnitude larger than what the historical pre‐colonial effective population size could have been in the same area. Considering that, on average, the *N*_*e*_ */N*_*c*_ ratio (Figure 3) is 0.21 (1950–2010), this reconstructed *N*_*e*_ > 5000 would represent a total population size exceeding 25,000 wolves, whereas prior to European settlement, the population size was rather 4000–7000 wolves for the entire region of Minnesota (based on an extrapolation of average pack size and home range).

VonHoldt et al. (2024) recently performed a similar analysis on the same population but based on a larger sample of wolves, including wolves from Michigan and Wisconsin and including the isolated Isle Royale population. Their recent *N*_*e*_ estimates were slightly smaller than ours (*N*_*e*_ = 228) but considering that they studied a population with a larger census size (*N*_tot_ fluctuating around 4000 instead of 2500 for Minnesota alone), these results require scrutiny. *N*_*e*_ estimation based on linkage disequilibrium assumes random mating across the entire population. Even weak population structure can downwardly bias estimates considerably (Ryman, Laikre, and Hössjer 2023). This can be avoided by sampling within a breeding window (the area within which mating in a continuous population can be considered random; Wright 1943) and making inferences on that breeding window alone (Neel et al. 2013). Our samples from Minnesota came from an area only slightly larger than a typical breeding window (45,000 km^2^; Mergeay et al. in press, albeit determined for a European population), whereas vonHoldt et al. (2024), covered a much larger area and included the isolated population of Isle Royale in Lake Superior. This likely increased linkage disequilibrium due to population structure (Neel et al. 2013) and led to an underestimation of the true *N*_*e*_, as also argued by Kardos and Waples (2024).

On top of this, the spatial scale at which *N*_*e*_ inferences are made when a subpopulation is targeted increases from the subpopulation to the total population the further the estimates go back in time (Novo et al. 2023a). The rate at which this happens depends on the effective size and the number of migrants.

Another layer of complexity is added when the number of migrants changes over time, which inevitably is a consequence of a change in the population size. Assuming a constant migration rate *m*, the number of migrants *N*_*m*_ a subpopulation receives depends on the size of the other populations. As the entire metapopulation in the larger region was nearly wiped out between 1800 and 1970, we can surmise that connectivity was also greatly affected and that our inferences during the 20th century largely reflect the effective size of the Minnesota population in isolation. We see the population reconstruction prior to 1900 increase, however, to an effective size of the pre‐colonial population exceeding 5000. This likely represents the part of an ancestral North American population in a much broader geographic region, of unknown extent (likely including the population in the adjacent states in the USA and Canada), and the march to equilibrium described above. Overall, we can make no meaningful quantitative deductions from this effective number of 5000–6000, other than that there was a large ancestral continuous or otherwise spatially structured metapopulation prior to European colonisation. Similar increases in ancestral *N*_*e*_ estimates numbering in the thousands were found by vonHoldt et al. (2024) across different wolf populations, indicating that this is not unique to this wolf population. This issue of increasing spatial scale as the size of a subpopulation within a structured larger population is reconstructed further back in time is common to other methods too, including those using the coalescent. This should not be confused with a true population decrease, unless there is certainty that the population is and always has been isolated (Novo et al. 2023a).

### 3.2 Scandinavian Peninsula Population, Europe

For the most recent cohort sample (2007–2014), GONE yields low *N*_*e*_ estimates that are slightly declining from the moment of founding (circa 1983) until the present, seemingly leading to a complete uncoupling of *N*_*e*_ and *N*_*c*_ (Figure S1). We failed to detect the severity of the founder effect (*N* = 2–3) and the subsequent steady population growth using this cohort. The lowest *N*_*e*_ estimated at any time is 26 (including migrant genotypes) or 23 (excluding migrant genotypes). As we track the *N*_*e*_ estimates back in time and we approach the time of the founding, we see the *N*_*e*_ estimates steadily rising, with values prior to the founding rising in the hundreds (Table 1).

**TABLE 1:** GONE *N*_*e*_ estimates of the Scandinavian wolf population for population cohorts sampled at different times in the past, along with the estimated year (assuming a 4‐year generation interval), and the mean number of packs across the 4‐year interval. For cohort 2007–2014, the first column includes all samples. In the second column 2007–2014 NI, four recent immigrant genotypes were excluded from the data set. Gen, generations ago.

Year	*N* packs	*N*_*e*_, 1983–1990	*N*_*e*_, 1991–1998	*N*_*e*_, 1999–2006	*N*_*e*_, 2007–2014	*N*_*e*_, 2007–2014 NI	Gen
2011	32.5				23	26	1
2007	20.2				23	26	2
2003	11			12	23	26	3
1999	7.8		11	12	23	26	4
1995	3		11	12	26	31	5
1991	1.5	6	11	12	28	29	6
1987	1	6	10	6	36	35	7
1983	0.5	6	6	6	51	41	8
1979	0	6	6	6	197	60	9
1975	0	23	10	7	295	107	10
1971	0	28	14	15	309	183	11
1967	0	28	14	18	320	274	12
1963	0	28	14	18	338	331	13
1959	0	36	21	22	363	337	14
1955	0	44	25	25	390	370	15

When we use the older cohort samples, however, we find for cohort 1999–2006 *N*_*e*_ = 12 for the last four generations, and a founder effect of *N*_*e*_ = 6 just before that, corresponding to the founding of the Scandinavian population. Similarly, for the two earlier cohorts (1991–1998, 1983–1990), GONE detects the same founder effect of *N*_*e*_ = 6 in the vicinity of the true time of the founding of the population (Table 1). The exact timing might be a bit off due to assumptions related to generation length. Even though this value still slightly overestimates the true founder effect of *N* = 3, it is very close. The failure to capture the true founder effect with the most recent cohort sample (2007–2014) did not depend on the inclusion or exclusion of the immigrant genotypes; the results are nearly identical for the past 10 generations. Excluding the four immigrant genotypes themselves does little to the genetic contribution of these immigrants to the other genotypes in the population. This discrepancy indicates that the net genetic contribution of the recent immigrants (2008 and 2013), which have a much more recent ancestry in a larger population, muddies the temporal reconstruction of the *N*_*e*_ of the Scandinavian population. Across the different temporal cohorts, the more ancient *N*_*e*_ reconstruction, prior to the founding of the Scandinavian population, differs by an order of magnitude. For the three earlier cohorts, the *N*_*e*_ estimates remain below 50. In contrast, the most recent cohort yielded *N*_*e*_ values around 1960 well in the hundreds. The Finnish–Karelian population, from which the population was founded, has remained small (hundreds of individuals) for over 150 years and has undergone a demographic and genetic bottleneck in the 20th century (Aspi et al. 2009; Jansson et al. 2012, 2014). A likely cause of this large difference in reconstructed *N*_*e*_ is that the most recent cohort includes the genetic contribution of three additional immigrants and their descendants in the Scandinavian population (Åkesson et al. 2022). Combined, these results call for caution in the interpretation of data when there is uncertainty with regard to how strong underlying model assumptions are met or violated. Here, it seems that violating the assumption of isolation (with realized gene flow in the 2007–2014 cohort) resulted in an upward bias in both the founding population size and the ancestral source population size (Table 1). The earlier cohorts were entirely descended from three founders and were not subjected to additional gene flow, and in those cases, GONE reconstructed the correct population trend, albeit with limited temporal resolution. However, even in isolated populations, known bottlenecks may not be retrieved by GONE (e.g., Cars et al. 2024), calling for caution with conclusions.

### 3.3 How Good Are the Most Recent *N*_*e*_ Estimates With GONE?

The current *N*_*e*_ we find for the last four generations in the Scandinavian population (*N*_*e*_ = 26) is low but reflects pedigree‐based simulations by Bruford (2015): For a simulated isolated population with an average size of 550 individuals, he found a decay of gene diversity across 10 generations of 11.3%. If we plug these numbers in *N*_*e*_ = −*t*/(2Ln(*H*_*t*_/*H*_0_)), and substitute *H*_10_/*H*_0_ with 0.887 (the fraction of gene diversity maintained across 10 generations), we get *N*_*e*_ = 41.5 for a population consisting of an average of 55 packs (using Svensson et al. (2023) to convert total population size to number of packs). The average number of packs between 2004 and 2014 was 28.7 (Svensson et al. 2023). Converting the *N*_*e*_/*N*_packs_ emerging from Bruford (2015) to the number of packs in 2007–2014, we get *N*_*e*_ = 22, which is remarkably close to the estimated value of *N*_*e*_ = 23. Considering that the number of packs is generally a good proxy for *N*_*e*_ in wolves (Mergeay et al. in press), it seems GONE's estimate for the most recent generations are robust.

Similarly, for the Minnesota population, we find recent *N*_*e*_ values that are of the same order of magnitude as the number of packs (GONE *N*_*e*_ = 284 for the most recent generations, mean *N*_packs_ 2012–2014 = 427). Note that this number of packs includes wolf pairs without offspring (Erb and Humpal 2022), whereas these are not included as packs in the Scandinavian monitoring (Svensson et al. 2023). If we consider that, in Scandinavia, on average 39% of territories are held by non‐reproductive pairs and apply this ratio to Minnesota, the corresponding number of packs excluding pairs is 260, which is close to the GONE estimate.

Similar studies on white‐tailed and mule deer also found a good correspondence between GONE *N*_*e*_ reconstructions and *N*_*c*_ in some (Kessler and Shafer 2024) but not all populations (Cars et al. 2024).

### 3.4 Further Testing of GONE?

GONE uses a very robust methodology to estimate past population size in natural populations. However, the *N*_*e*_ calculated from linkage disequilibrium among pairs of loci depends on numerous assumptions (Neel et al. 2013; Ryman, Laikre, and Hössjer 2019), among which random mating is a really important one, and by extension an absence of spatial genetic structure in the population for which the estimate is made. Very few natural populations fulfil this assumption, but there are ways to cope with this issue, at least for contemporary point estimates (e.g., Mergeay et al. in press), as the sum of subpopulation *N*_*e*_ estimates is a good proxy of the metapopulation *N*_*e*_, at least in well‐connected subpopulations (Spieth 1974; Ryman, Laikre, and Hössjer 2019). In spatially structured populations in which subpopulations exchange migrants, sampling individual subpopulations (fulfilling by themselves the random mating assumption) yields at first contemporary *N*_*e*_ estimates of each individual subpopulation. As the estimates go back in time for earlier generations, the estimates are increasingly affected by the metapopulation *N*_*e*_ (Novo et al. 2023a) and the values at some time represent an intermediate between the local and the meta *N*_*e*_ before converging to the meta *N*_*e*_. Without detailed information on gene flow and the metapopulation, it is hard to know what the meaning of this intermediate value is. Moreover, in populations that underwent declines or expansions, there is a confounding signal of demographic change that becomes hard if not impossible to disentangle from this transition from local to metapopulation *N*_*e*_. Although GONE is a game changer in the estimation of effective population sizes, we need to remain cautious with the output, and thoroughly explore to what extent the results might be affected by the violation of assumptions of which we have incomplete knowledge. Further empirical comparisons between reconstructed *N*_*e*_ and known demographies in species with other traits and life histories (such as Novo et al. 2023b; Kessler and Shafer 2024; Cars et al. 2024) will improve our knowledge on the limits of the method.

## 4 Conclusions

Overall, we find that GONE was capable of reconstructing wolf population trends relatively well, within certain limits. The Minnesota data set originates from a relatively isolated well‐defined population that has not been subjected to significant gene flow in recent (20) generations, thereby adhering to a major assumption of the method. As a result, the *N*_*e*_ reconstruction matches the historical demography, even though the reconstructed *N*_*e*_/*N*_*c*_ ratio, in as much as it can be considered accurate, was not entirely constant. We cannot, however, disentangle the change in demography with the effect of connectivity in a large metapopulation prior to 1900. Without prior knowledge of the regional context, we would falsely interpret the decline from *N*_*e*_ = 5000 to the present *N*_*e*_ = 284 as a 95% reduction in population size and consider the population is doing poorly. The Scandinavian wolf data set highlighted that recent gene flow can rapidly obscure recent demographic details. There we needed the historical samples to retrieve the 1980s founder effect. Overall, GONE seems a powerful tool, but like all methods, it needs to be used with caution and results need to be interpreted with care.

## Conflicts of Interest

The authors declare no conflicts of interest.

## Supporting information


Appendix S1.

## Data Availability

The analyses carried out in this study and the related scripts are available at: https://github.com/villarpau/Wolf_Ne_reconstructions.
