# Supplementary material for: Comparing Genetic Ne Reconstructions Over Time With Long‐Time Wolf Monitoring Data in Two Populations
Source: Evol Appl. 2024 Oct 17;17(10):e70022. doi: 10.1111/eva.70022 (PMC11486914; doi:10.1111/eva.70022)
Supplement: Supplementary file 1 — Appendix S1. [file EVA-17-e70022-s001.docx]

Table S1. Metadata available for Minnesota samples used in the study (Modified from Cronin et al., 2014).

| Specimen number | Country | U.S. State | Tissue | Year | Collector |
| --- | --- | --- | --- | --- | --- |
| 20 | St. Louis County | Minnesota | muscle in ETOH | 2012 | Carolin Humpal MN DNR |
| 21 | St. Louis County | Minnesota | muscle in ETOH | 2012 | Carolin Humpal MN DNR |
| 22 | St. Louis County | Minnesota | muscle in ETOH | 2012 | Carolin Humpal MN DNR |
| 23 | Lake County | Minnesota | muscle in ETOH | 2012 | Carolin Humpal MN DNR |
| 24 | Itasca County | Minnesota | muscle in ETOH | 2012 | Carolin Humpal MN DNR |
| 25 | Pine County | Minnesota | muscle in ETOH | 2012 | Carolin Humpal MN DNR |
| 26 | St. Louis County | Minnesota | muscle in ETOH | 2012 | Carolin Humpal MN DNR |
| 27 | Lake of the Woods Co | Minnesota | muscle in ETOH | 2012 | Carolin Humpal MN DNR |
| 28 | Kittson County | Minnesota | muscle in ETOH | 2012 | Carolin Humpal MN DNR |
| 29 | Otter Trail | Minnesota | muscle in ETOH | 2012 | Carolin Humpal MN DNR |
| 30 | Beltrami County | Minnesota | muscle in ETOH | 2012 | Carolin Humpal MN DNR |
| 31 | Hubbard County | Minnesota | muscle in ETOH | 2012 | Carolin Humpal MN DNR |
| 32 | Cass County | Minnesota | muscle in ETOH | 2012 | Carolin Humpal MN DNR |
| 33 | Cook County | Minnesota | muscle in ETOH | 2012 | Carolin Humpal MN DNR |
| 34 | Kanabec County | Minnesota | muscle in ETOH | 2012 | Carolin Humpal MN DNR |
| 35 | Hubbard County | Minnesota | muscle in ETOH | 2012 | Carolin Humpal MN DNR |
| 36 | Cass County | Minnesota | muscle in ETOH | 2012 | Carolin Humpal MN DNR |
| 37 | Koochiching County | Minnesota | muscle in ETOH | 2012 | Carolin Humpal MN DNR |
| 38 | Polk County | Minnesota | muscle in ETOH | 2012 | Carolin Humpal MN DNR |
| 39 | Beltrami County | Minnesota | muscle in ETOH | 2013 | Carolin Humpal MN DNR |

Table S2. Metadata available for Scandinavian samples used in the study (Modified from Smeds and Ellegren, 2022).

| **UU_ID** | **ENA_ID** | **Population** | **Category** | **GenClass** | **Sex** | **X Coverage** | **SRA Accession** | **Citation** |
| --- | --- | --- | --- | --- | --- | --- | --- | --- |
| 16-D-89-03 | D-89-03 | Scandinavia | 1983-1990 | F2 | female | 33 | ERS1754904 | Kardos et al., 2018 |
| 17-D-92-05 | D-92-05 | Scandinavia | 1983-1990 | F2 | male | 37 | ERS1754908 | Kardos et al., 2018 |
| 19-D-94-01 | D-94-01 | Scandinavia | 1983-1990 | F3 | female | 26 | ERS1754913 | Kardos et al., 2018 |
| 30-D-93-02 | D-93-02 | Scandinavia | 1983-1990 | F2 | female | 33 | ERS1754911 | Kardos et al., 2018 |
| 31-D-93-03 | D-93-03 | Scandinavia | 1983-1990 | F2 | female | 22 | ERS1754912 | Kardos et al., 2018 |
| 3-D-86-01 | D-86-01 | Scandinavia | 1983-1990 | F1 | male | 37 | ERS1754902 | Kardos et al., 2018 |
| 4-D-89-01 | D-89-01 | Scandinavia | 1983-1990 | F2 | male | 32 | ERS1754903 | Kardos et al., 2018 |
| 57-D-92-06 | D-92-06 | Scandinavia | 1983-1990 | F3 | male | 39 | ERS1754909 | Kardos et al., 2018 |
| 5-D-91-01 | D-91-01 | Scandinavia | 1983-1990 | F2 | female | 30 | ERS1754905 | Kardos et al., 2018 |
| 6-M-98-02 | M-98-02 | Scandinavia | 1983-1990 | F2 | male | 30 | ERS1754962 | Kardos et al., 2018 |
| D-84-03 | D-84-03 | Scandinavia | 1983-1990 | F1 | male | 45 | ERS1754899 | Kardos et al., 2018 |
| D-85-02 | D-85-02 | Scandinavia | 1983-1990 | F1 | male | 42 | ERS1754901 | Kardos et al., 2018 |
| 105-D-00-15 | D-00-15 | Scandinavia | 1991-1998 | F2 | male | 32 | ERS1754869 | Kardos et al., 2018 |
| 11-M-98-03 | M-98-03 | Scandinavia | 1991-1998 | F1 | female | 28 | ERS1754963 | Kardos et al., 2018 |
| 12-M-01-04 | M-01-04 | Scandinavia | 1991-1998 | F2 | female | 15 | ERS1754943 | Kardos et al., 2018 |
| 13-M-98-08 | M-98-08 | Scandinavia | 1991-1998 | F2 | male | 38 | ERS1754964 | Kardos et al., 2018 |
| 14-M-00-09 | M-00-09 | Scandinavia | 1991-1998 | F2 | male | 45 | ERS1754941 | Kardos et al., 2018 |
| 15-M-01-10 | M-01-10 | Scandinavia | 1991-1998 | F3 | female | 36 | ERS1754945 | Kardos et al., 2018 |
| 22-D-99-02 | D-99-02 | Scandinavia | 1991-1998 | F1 | male | 34 | ERS1754915 | Kardos et al., 2018 |
| 28-M-03-07 | M-03-07 | Scandinavia | 1991-1998 | F3 | female | 34 | ERS1754948 | Kardos et al., 2018 |
| 35-D-05-23 | D-05-23 | Scandinavia | 1991-1998 | F3 | male | 33 | ERS1754872 | Kardos et al., 2018 |
| 43-D-92-02 | D-92-02 | Scandinavia | 1991-1998 | F1 | male | 29 | ERS1754907 | Kardos et al., 2018 |
| 59-D-96-01 | D-96-01 | Scandinavia | 1991-1998 | F1 | male | 34 | ERS1754914 | Kardos et al., 2018 |
| 94-D-92-01 | D-92-01 | Scandinavia | 1991-1998 | F1 | male | 35 | ERS1754906 | Kardos et al., 2018 |
| D-01-18 | D-01-18 | Scandinavia | 1991-1998 | F2 | male | 30 | ERS1754870 | Kardos et al., 2018 |
| D-93-01 | D-93-01 | Scandinavia | 1991-1998 | F1 | male | 40 | ERS1754910 | Kardos et al., 2018 |
| M-98-01 | M-98-01 | Scandinavia | 1991-1998 | F3 | female | 34 | ERS1754961 | Kardos et al., 2018 |
| 103-D-11-17 | D-11-17 | Scandinavia | 1999-2006 | F4 | female | 28 | ERS1754894 | Kardos et al., 2018 |
| 106-D-10-20 | D-10-20 | Scandinavia | 1999-2006 | F2 | female | 30 | ERS1754886 | Kardos et al., 2018 |
| 109-D-10-30 | D-10-30 | Scandinavia | 1999-2006 | F3 | male | 17 | ERS1754889 | Kardos et al., 2018 |
| 110-M-01-06 | M-01-06 | Scandinavia | 1999-2006 | F3 | female | 23 | ERS1754944 | Kardos et al., 2018 |
| 112-D-10-29 | D-10-29 | Scandinavia | 1999-2006 | F3 | male | 24 | ERS1754888 | Kardos et al., 2018 |
| 25-M-03-06 | M-03-06 | Scandinavia | 1999-2006 | F2 | male | 41 | ERS1754947 | Kardos et al., 2018 |
| 36-D-06-14 | D-06-14 | Scandinavia | 1999-2006 | F3 | female | 37 | ERS1754873 | Kardos et al., 2018 |
| 37-M-06-03 | M-06-03 | Scandinavia | 1999-2006 | F4 | male | 35 | ERS1754951 | Kardos et al., 2018 |
| 38-D-06-16 | D-06-16 | Scandinavia | 1999-2006 | F3 | female | 33 | ERS1754874 | Kardos et al., 2018 |
| 39-M-06-04 | M-06-04 | Scandinavia | 1999-2006 | F4 | female | 24 | ERS1754952 | Kardos et al., 2018 |
| 40-D-07-28 | D-07-28 | Scandinavia | 1999-2006 | F3 | male | 30 | ERS1754879 | Kardos et al., 2018 |
| 45-D-07-24 | D-07-24 | Scandinavia | 1999-2006 | F3 | female | 15 | ERS1754878 | Kardos et al., 2018 |
| 51-D-07-09 | D-07-09 | Scandinavia | 1999-2006 | F5 | male | 19 | ERS1754875 | Kardos et al., 2018 |
| 52-D-07-17 | D-07-17 | Scandinavia | 1999-2006 | F5 | female | 20 | ERS1754877 | Kardos et al., 2018 |
| 53-D-08-08 | D-08-08 | Scandinavia | 1999-2006 | F5 | female | 24 | ERS1754880 | Kardos et al., 2018 |
| 54-M-07-06 | M-07-06 | Scandinavia | 1999-2006 | F4 | male | 18 | ERS1754954 | Kardos et al., 2018 |
| 55-D-08-10 | D-08-10 | Scandinavia | 1999-2006 | F3 | female | 14 | ERS1754881 | Kardos et al., 2018 |
| 60-M-00-10 | M-00-10 | Scandinavia | 1999-2006 | F3 | male | 37 | ERS1754942 | Kardos et al., 2018 |
| 75-G9-05 | G9-05 | Scandinavia | 1999-2006 | F2 | male | 17 | ERS1754939 | Kardos et al., 2018 |
| 80-M-09-17 | M-09-17 | Scandinavia | 1999-2006 | F2 | male | 13 | ERS1754957 | Kardos et al., 2018 |
| 81-M-09-05 | M-09-05 | Scandinavia | 1999-2006 | F3 | male | 13 | ERS1754956 | Kardos et al., 2018 |
| D-00-12 | D-00-12 | Scandinavia | 1999-2006 | F3 | female | 28 | ERS1754868 | Kardos et al., 2018 |
| D-08-20 | D-08-20 | Scandinavia | 1999-2006 | F4 | male | 41 | ERS1754883 | Kardos et al., 2018 |
| M-05-07 | M-05-07 | Scandinavia | 1999-2006 | F4 | female | 39 | ERS1754950 | Kardos et al., 2018 |
| 100-G47-11 | G47-11 | Scandinavia | 2007-2014I | L1 | female | 34 | ERS1754933 | Kardos et al., 2018 |
| 102-G34-10 | G34-10 | Scandinavia | 2007-2014S | F4 | female | 34 | ERS1754931 | Kardos et al., 2018 |
| 104-G100-14 | G100-14 | Scandinavia | 2007-2014I | L1 | male | 36 | ERS1754917 | Kardos et al., 2018 |
| 107-D-10-53 | D-10-53 | Scandinavia | 2007-2014I | L1 | male | 21 | ERS1754892 | Kardos et al., 2018 |
| 108-D-10-44 | D-10-44 | Scandinavia | 2007-2014S | F6 | male | 24 | ERS1754890 | Kardos et al., 2018 |
| 111-G110-11 | G110-11 | Scandinavia | 2007-2014S | F4 | female | 29 | ERS1754920 | Kardos et al., 2018 |
| 48-D-08-21 | D-08-21 | Scandinavia | 2007-2014S | F3 | male | 15 | ERS1754884 | Kardos et al., 2018 |
| 56-D-07-16 | D-07-16 | Scandinavia | 2007-2014S | F3 | male | 24 | ERS1754876 | Kardos et al., 2018 |
| 61-G97-13 | G97-13 | Scandinavia | 2007-2014S | F3 | male | 27 | ERS1754940 | Kardos et al., 2018 |
| 62-G174-13 | G174-13 | Scandinavia | 2007-2014S | F5 | female | 37 | ERS1754924 | Kardos et al., 2018 |
| 65-M-10-04 | M-10-04 | Scandinavia | 2007-2014I | L1 | female | 16 | ERS1754958 | Kardos et al., 2018 |
| 67-M-11-02 | M-11-02 | Scandinavia | 2007-2014I | L1 | female | 14 | ERS1754960 | Kardos et al., 2018 |
| 68-D-10-68 | D-10-68 | Scandinavia | 2007-2014S | F4 | male | 13 | ERS1754893 | Kardos et al., 2018 |
| 69-D-10-15 | D-10-15 | Scandinavia | 2007-2014S | F6 | female | 15 | ERS1754885 | Kardos et al., 2018 |
| 70-D-10-23 | D-10-23 | Scandinavia | 2007-2014S | F6 | female | 15 | ERS1754887 | Kardos et al., 2018 |
| 73-D-11-22 | D-11-22 | Scandinavia | 2007-2014S | F4 | female | 11 | ERS1754895 | Kardos et al., 2018 |
| 76-G109-11 | G109-11 | Scandinavia | 2007-2014I | L2 | male | 12 | ERS1754919 | Kardos et al., 2018 |
| 77-G87-12 | G87-12 | Scandinavia | 2007-2014S | F5 | female | 9 | ERS1754938 | Kardos et al., 2018 |
| 78-D-11-58 | D-11-58 | Scandinavia | 2007-2014S | F4 | female | 11 | ERS1754896 | Kardos et al., 2018 |
| 84-G37-10 | G37-10 | Scandinavia | 2007-2014I | L1 | male | 13 | ERS1754932 | Kardos et al., 2018 |
| 85-G100-12 | G100-12 | Scandinavia | 2007-2014I | L2 | male | 13 | ERS1754916 | Kardos et al., 2018 |
| 86-G126-13 | G126-13 | Scandinavia | 2007-2014S | F5 | male | 12 | ERS1754922 | Kardos et al., 2018 |
| 87-G139-12 | G139-12 | Scandinavia | 2007-2014I | L2 | female | 11 | ERS1754923 | Kardos et al., 2018 |
| 88-G50-12 | G50-12 | Scandinavia | 2007-2014S | F4 | male | 11 | ERS1754934 | Kardos et al., 2018 |
| 89-G67-15 | G67-15 | Scandinavia | 2007-2014I | L1 | female | 23 | ERS1754936 | Kardos et al., 2018 |
| 91-G24-14 | G24-14 | Scandinavia | 2007-2014S | F5 | female | 9 | ERS1754927 | Kardos et al., 2018 |
| 92-G58-15 | G58-15 | Scandinavia | 2007-2014S | F5 | female | 25 | ERS1754935 | Kardos et al., 2018 |
| 95-G175-13 | G175-13 | Scandinavia | 2007-2014S | F4 | male | 39 | ERS1754925 | Kardos et al., 2018 |
| 96-G111-14 | G111-14 | Scandinavia | 2007-2014I | L2 | male | 19 | ERS1754921 | Kardos et al., 2018 |
| 97-G32-12 | G32-12 | Scandinavia | 2007-2014S | F3 | male | 24 | ERS1754929 | Kardos et al., 2018 |
| 98-G32-15 | G32-15 | Scandinavia | 2007-2014I | L3 | female | 37 | ERS1754930 | Kardos et al., 2018 |
| 99-G106-13 | G106-13 | Scandinavia | 2007-2014I | L2 | male | 34 | ERS1754918 | Kardos et al., 2018 |
| D-08-19 | D-08-19 | Scandinavia | 2007-2014S | F4 | female | 29 | ERS1754882 | Kardos et al., 2018 |
| D-10-50 | D-10-50 | Scandinavia | 2007-2014S | F4 | male | 21 | ERS1754891 | Kardos et al., 2018 |
| W89r | W89 | Scandinavia | 2007-2014I | L3 | male | 24 | ERS2672925 | Smeds et al. 2019 |
| W99r | W99 | Scandinavia | 2007-2014I | L3 | male | 30 | ERS2672936 | Smeds et al. 2019 |
| W9r | W9 | Scandinavia | 2007-2014I | L2 | male | 24 | ERS2672926 | Smeds et al. 2019 |
| 63-M-10-10 | M-10-10 | Scandinavia | R_immigrants | - | male | 38 | ERS1754959 | Kardos et al., 2018 |
| 79-M-09-03 | M-09-03 | Scandinavia | R_immigrants | - | male | 30 | ERS1754955 | Kardos et al., 2018 |
| 82-G23-13 | G23-13 | Scandinavia | R_immigrants | - | male | 12 | ERS1754926 | Kardos et al., 2018 |
| 83-G31-13 | G31-13 | Scandinavia | R_immigrants | - | female | 12 | ERS1754928 | Kardos et al., 2018 |


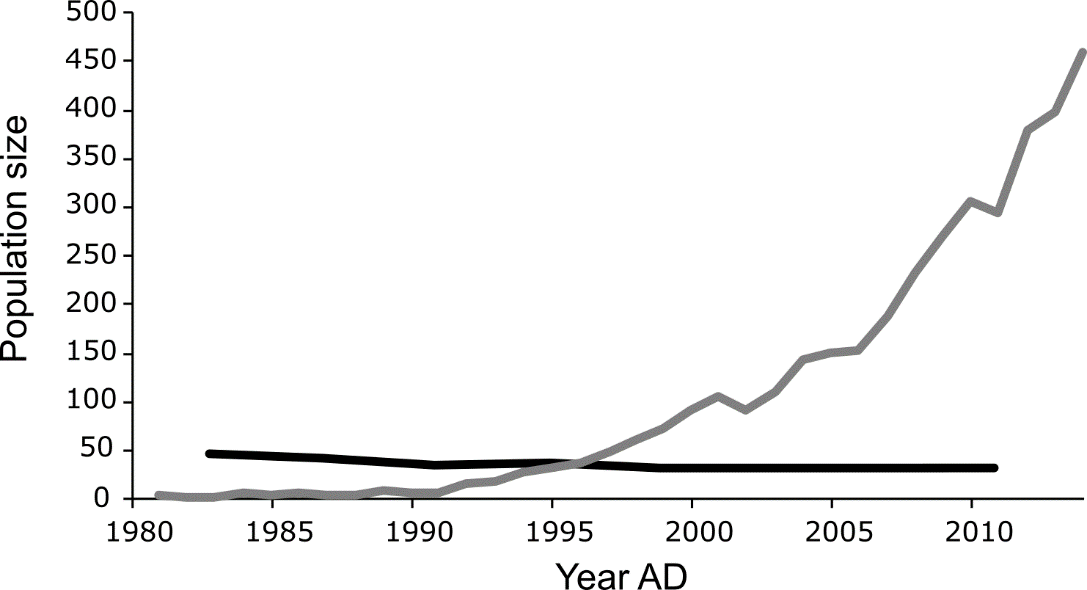


Figure S1. Detailed comparison of *N_e_* reconstructed from the 2007-2014 cohort without immigrants (black line) using GONE, and total number of wolves (N_tot_) for the period 1981 to 2014 (grey line). Total population size was taken from Wabakken et al. 2001 (1981-2000) and <https://www.slu.se/centrumbildningar-och-projekt/viltskadecenter/publikationer/inventeringsrapporter/inventeringsrapporter-varg/> (2000-2014).
